# Clinical Approach to STRIDE-II in Real-Life Settings: Analysis and Practical Recommendations

**DOI:** 10.1093/crocol/otae055

**Published:** 2024-10-10

**Authors:** Elena Ricart, Guillermo Bastida, Daniel Carpio, Daniel Ceballos, Daniel Ginard, Ignacio Marín-Jimenéz, Luis Menchén, Fernando Muñoz, Yago González-Lama

**Affiliations:** Inflammatory Bowel Disease Unit, Gastroenterology Department, Hospital Clinic Barcelona, IDIBAPS, CIBEREHD, Barcelona 08036, Spain; Gastroenterology Department, La Fe University and Polytechnic Hospital, Valencia 46026, Spain; Gastroenterology Department, Complexo Hospitalario Universitario de Pontevedra, Instituto de Investigación Sanitaria Galicia Sur (IISGS), Pontevedra 36071, Spain; Gastroenterology Department, Hospital Universitario Doctor Negrin, Las Palmas de Gran Canaria 35010, Spain; Gastroenterology Department, Hospital Universitario Son Espases, Palma de Mallorca 07120, Spain; Gastroenterology Department, Departamento de Medicina, Facultad de Medicina, Universidad Complutense de Madrid, Hospital Universitario Gregorio Marañón-Instituto de Investigación Sanitaria Gregorio Marañón, Madrid 28007, Spain; Gastroenterology Department, Departamento de Medicina, Facultad de Medicina, Universidad Complutense de Madrid, Hospital Universitario Gregorio Marañón-Instituto de Investigación Sanitaria Gregorio Marañón, Madrid 28007, Spain; Gastroenterology Department, Hospital Universitario de Salamanca, Salamanca 37007, Spain; Inflammatory Bowel Disease Unit, Gastroenterology Department, Hospital Universitario 12 de Octubre, Madrid 28041, Spain

**Keywords:** inflammatory bowel disease, Crohn’s disease, ulcerative colitis, implementation, expert opinions

## Abstract

**Background:**

We aimed to (1) analyze the applicability of the updated Selecting Therapeutic Targets in Inflammatory Bowel Disease (STRIDE-II) recommendations in real-world clinical practice, (2) identify barriers to their implementation, and (3) propose practical measures to overcome these obstacles.

**Methods:**

This qualitative study was based on a survey, a literature review, and expert opinions. Nine inflammatory bowel disease (IBD) experts identified 7 areas likely to be controversial or potential implementation barriers in daily clinical practice: endoscopy, histology, ultrasound, quality of life, biomarkers, symptom control, and patient-reported outcomes (PROs). Based on this, a survey was carried out among educational course participants. The experts discussed the literature review and survey results and proposed several statements and practical actions.

**Results:**

A total of 55 gastroenterologists answered the survey. The reported difficulty level in reaching STRIDE-II treatment goals in clinical practice was high. Only 22% of participants performed clinical remission assessments using clinical indexes and PROs. Seventy percent of responders did not use fecal calprotectin cutoffs and considered changes from the previous levels instead. Mucosal healing as a long-term therapeutic goal was considered necessary to be individualized in specific patient subgroups (eg, elderly/fragile patients, multiple treatment failures, and last-line therapies). Other barriers, like the lack of access to imaging techniques or insufficient knowledge and skills among healthcare professionals, were detected. The experts suggested adding less stringent treatment goals and measurements, patient stratification, local adaptations, educational activities, and research.

**Conclusions:**

STRIDE-II recommendations face various implementation barriers needing careful evaluation in order to enhance their adoption in clinical practice, and ultimately improve outcomes in IBD patients.

## Graphical Abstract

**Graphical Abstract:**
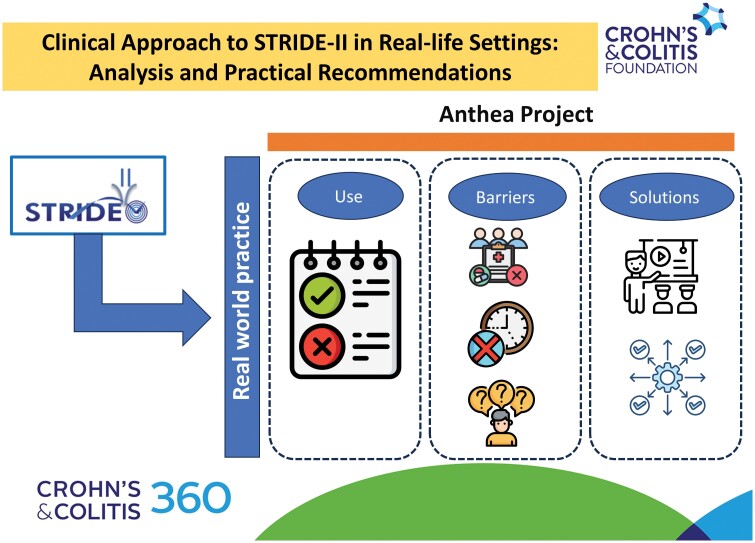

## Introduction

Crohn’s disease (CD) and ulcerative colitis (UC) are inflammatory bowel diseases (IBDs) with a significant impact on patients, society, and health systems.^1,2^

New insights into disease pathophysiology, availability of advanced diagnostic tools and biomarkers, and new therapy approval have improved short- and long-term patient outcomes.^3–5^

Therefore, there is a great interest in defining and measuring treatment goals and strategies in the current context.^6^ Before the advent of advanced therapies (biologics and small molecules), IBD treatment goals mainly focused on symptom control, steroid avoidance, and surgery prevention. However, the availability of more effective drugs led to the inclusion of more ambitious goals such as patient-centered outcomes (eg, restoring and maintaining the quality of life and work productivity) and objective disease control markers like endoscopic, histological, or transmural healing.^7,8^ Moreover, different treatment strategies have emerged, such as combination therapy, top-down approaches, and treat-to-target strategies.^9,10^

In 2015, the Selecting Therapeutic Targets in Inflammatory Bowel Disease (STRIDE) initiative, proposed evidence- and consensus-based IBD treatment targets that could be used in treat-to-target strategies in routine clinical practice.^11^ The 2021 update (STRIDE-II) includes 13 recommendations incorporating time-dependent treatment targets (short-term, intermediate, and long-term) and drug-specific time points for treat-to-target strategies in adults and children with IBD.^6^

However, concerns were expressed regarding the validity, applicability, and impact on daily practice of these recommendations.^12^ Recently, the IBD-PODCAST cross-sectional study stated that objective data for IBD evaluation (included in STRIDE-II) from daily clinical practice were only available in a limited number of patients. This suggests that, at least in part, guideline application might be suboptimal. Moreover, treatment goals like mucosal healing, which, depending on the definition and selected therapy, are not achieved in many patients, have proved controversial.^13^

The Analyzing Therapeutic Objectives in IBD (ANTHEA) project was designed to (1) analyze the current application of STRIDE-II recommendations in real-world clinical practice, (2) identify controversies and potential obstacles to the implementation of these guidelines, and (3) propose practical actions to overcome these barriers.

## Materials and Methods

### Study Design

The ANTHEA project was designed to study the use of STRIDE-II recommendations, including the barriers to their implementation, and to propose practical actions and strategies to overcome them. This project was developed in several steps, including an in-person meeting with an anonymous survey, a comprehensive literature review, and an IBD expert discussion group.

### Ethical Considerations

This study was conducted using the Good Clinical Practice guidelines and the current version of the revised World Medical Association’s Declaration of Helsinki.

### In-Person Meeting

First, a steering committee (SC) composed of nine gastroenterologists with IBD expertise was established. The SC analyzed the 2021 STRIDE-II^6^ recommendations and identified 7 areas that might present implementation problems in daily practice, including symptom control, patient-reported outcomes (PROs), endoscopy, histology, imaging techniques, quality of life, and biomarkers. A mixed (online and in-person) meeting was designed based on these observations. In the first part, the participants accessed an online platform for which the SC recorded 4 videos and posted relevant scientific articles dealing with these controversial topics. The course participants reviewed these information materials before the in-person session. After that, 6 in-person meetings were organized in different cities across the country during which participants, under the guidance of SC members, discussed the topics and suggested solutions.

### Survey

All participants completed a structured, anonymized, and closed survey at the end of the in-person meeting. The survey was divided into 2 main sections including different questions and variables: (1) sociodemographic and medical practice-related variables (age, years of clinical practice, and experience in IBD patient management); (2) opinion and attitude in daily practice related to the STRIDE-II treatment goals and others, including the difficulty level to achieve them at any time in IBD patients (from 1 = minimum difficulty to 10 = maximum difficulty); importance level of short-term treatment goals (from 1 = the most important to 9 or 8 = the least important in CD and UC, respectively); clinical remission measurement (clinical indexes, PROs, etc.); fecal calprotectin (FC), (time to assessment and cutoff values); mucosal healing (time to assessment, magnetic resonance enterography [MRE], patient profile, activity indexes); histological remission in UC; transmural healing in CD; quality of life, and well-being.

### Literature Review

A narrative literature review was carried out in Medline with the help of an experienced documentalist. We used PubMed’s Clinical Queries tool and individual searches using MeSH and free-text terms up to February 2024. The aim of our search was to identify articles on adults with IBD analyzing STRIDE-II recommendation implementation and use in daily practice. Meta-analyses, systematic literature reviews (SLRs), randomized controlled trials (RCTs), and observational studies were included. Two reviewers independently selected articles, first by title and abstract, then by reading the full articles, and collected data. Evidence and result tables were generated. Study quality was assessed using the 2011 Oxford scale.^14^

### IBD Expert Discussion Group

The SC discussed the survey and literature review results. They proposed several statements, strategies, and recommendations for a rational and practical approach to STRIDE-II consensus^6^ from a real-world clinical practice perspective.

### Statistical Analysis

A descriptive analysis of the survey was performed. Depending on data distribution, frequency distribution, mean and standard deviation, or median and interquartile range were employed. Analyses were performed using Stata 12 statistical software (Stata Corporation, College Station, TX, USA).

### Final Document

The final document was written based on the survey results, literature review, and discussions among experts. The document was sent to SC members for final assessment and comments.

## Results

### Survey

A total of 55 gastroenterologists completed the survey (Table 1). Nearly 50% reported a clinical experience of more than 10 years, and 68% regularly monitored IBD patients in a dedicated IBD clinic consultation or unit.

**Table 1.** Main characteristics of the survey participants^a^.

	*n* (%)
Gastroenterologist (*n* = 55)	
Specialist	45 (82%)
Trainee	10 (18%)
Work experience	
<5 years	20 (36%)
5-10 years	10 (18%)
10-20 years	14 (26%)
> 20 years	11 (20%)
Type of consultation for IBD patients	
General consultation (many IBD patients)	3 (5%)
General consultation (few IBD patients)	15 (27%)
Monographic IBD clinic 1-2 days/week	31 (57%)
Monographic IBD unit	6 (11%)

Abbreviation: IBD, inflammatory bowel disease.

^a^Results are expressed as numbers and percentages (%); otherwise, it is indicated.

Regarding the STRIDE-II treatment goals, the difficulty of achieving them in daily practice in IBD patients (from 1 = minimum to 10 = maximum difficulty) was generally quite challenging (Table 2). The most feasible treatment goal was clinical remission, followed by surgery avoidance, with a mean of 4.27 ± 2.16 and 4.55 ± 2.01, respectively. Conversely, transmural healing, with a mean of 7.24 ± 1.82, and histological remission, with a mean of 7.22 ± 1.64, were the most difficult to achieve.

**Table 2.** The dfficulty of achieving the STRIDE-II proposed treatment goals and others in daily practice in patients with IBD (1 = minimum difficulty to 10 = maximum difficulty)^a^.

Treatment goal	Difficulty
Clinical remission	4.27 (2.16)
Avoidance of surgery	4.55 (2.01)
Normalization of serum and fecal inflammatory biomarkers	4.82 (1.86)
Absence of disability	4.93 (1.92)
Normalization of quality of life	5.40 (1.76)
Mucosal healing	5.95 (1.72)
Absence of fatigue	6.00 (2.34)
Histologic remission	7.22 (1.64)
Transmural remission	7.24 (1.82)

Abbreviation: IBD, inflammatory bowel disease.

^a^Results are expressed as mean (standard deviation).

When ranking the importance level of STRIDE-II treatment goals in the short or medium term (Table 3), we observed that the most important goal was clinical response in CD patients (as reported by 51% of responders), closely followed by clinical remission (for 44% of participants). The least important ones were histological remission and transmural healing (for 51% and 40% of participants). Similarly, for UC patients, 69% of responders indicated that clinical response was the most important treatment goal, followed by clinical remission (22%). Conversely, histological remission and absence of disability were ranked as the least important goals by 64% and 25% of gastroenterologists, respectively.

**Table 3.** Ranking of the importance of short-medium term treatment goals in IBD (mean scores and standard deviation).

Crohn disease		Ulcerative colitis	
Goals (order of importance)^a^	Score	Goals (order of importance)^b^	Score
Clinical remission	1.65 (0.73)	Clinical response	1.80 (1.65)
Clinical response	1.95 (1.46)	Clinical remission	1.96 (0.72)
Biomarkers normalization	3.95 (1.39)	Biomarkers normalization	3.98 (1.28)
Endoscopic response	4.64 (1.28)	Endoscopic response	4.71 (1.33)
Normalization of quality of life	5.20 (1.78)	Normalization of quality of life	4.76 (1.77)
Endoscopic remission	5.47 (1.46)	Endoscopic remission	5.55 (1.49)
Absence of disability	5.89 (2.02)	Absence of disability	5.78 (2.00)
Transmural remission	8.04 (1.15)	Histologic remission	7.45 (0.86)
Histologic remission	8.22 (0.98)	-	-

Abbreviation: IBD, inflammatory bowel disease.

^a^1, Most Important; 9, Least Important.

^b^1, Most Important; 8, Least Important.

Regarding clinical remission assessment, 49% of participants reported using clinical indexes in daily practice but not PROs, whereas 22% used both. Besides, almost a quarter of gastroenterologists did not apply validated clinical indexes or PROs to evaluate clinical remission.

Fecal calprotectin use was extensively analyzed (Table 4). A survey question examined the timing of the first FC assessment once the treatment was started. Many responders noted that FC was initially assessed 8 or 12 weeks after treatment initiation in patients with ileal CD and UC (according to 67% and 56% of participants, respectively). Another question assessed the cutoff values considered good enough after treatment initiation. However, 70% of gastroenterologists reported a lack of clear FC cutoffs and instead considered changes from previous levels (total reductions).

**Table 4.** Fecal calprotectin assessment in patients with IBD^a^.

	Ileal Crohn’s disease	Ulcerative colitis
Time to assessment after treatment initiation		
2 weeks	1 (2%)	2 (4%)
4 weeks	7 (13%)	19 (35%)
8 weeks	22 (40%)	22 (40%)
12 weeks	15 (27%)	9 (16%)
After induction and only if remission is not achieved	10 (18%)	3 (5%)
Cutoff value to consider it sufficient		
<250 µg/g	5 (10%)	8 (15%)
<150 µg/g	9 (16%)	8 (15%)
<100 µg/g	2 (4%)	0 (0%)
I do not have a clear cutoff and analyze changes from the previous level (total reductions)	39 (70%)	39 (70%)

Abbreviations: g, gram; IBD, inflammatory bowel disease; µg, microgram.

^a^Results are expressed as numbers and percentages (%).

Three questions were related to mucosal healing. As shown in Table 5, except for 1 gastroenterologist, all patients with ileal CD underwent intestinal evaluation after treatment initiation, regardless of the patient’s clinical remission status, with 69% of gastroenterologists preferring MRE over endoscopy. Conversely, in UC patients, 44% of participants performed an endoscopic evaluation 6-12 months after treatment initiation, while 35% did not perform it in case of clinical remission. Mucosal healing applicability as a long-term treatment goal was analyzed using specific patient profiles. Most responders agreed that this should carefully be individualized (even setting it aside if necessary), especially in elderly/fragile patients, in cases of multiple treatment failure and last-line therapies since these subgroups of patients often present with unique clinical challenges—such as increased comorbidities, reduced tolerance to therapies, and limited treatment options—which necessitate adjusted therapeutic goals to ensure safety and quality of life rather than complete mucosal healing. Forty-seven gastroenterologists (85%) reported using endoscopic clinical indexes in daily practice. However, 19 (35%) expressed concerns about the reliability of general endoscopists who are not specialized in IBD, sometimes leading them to neglect endoscopic findings. Additionally, 10 gastroenterologists (18%) deemed endoscopic cutoff values excessively stringent for daily practice.

**Table 5.** Time to mucosal healing assessment in patients with IBD after treatment initiation^a^.

Time to assessment	Ileal Crohn disease	Ulcerative colitis
No, if the patient is in clinical remission	1 (2%)	19 (35%)
3 months	2 (4%)	2 (4%)
6-12 months	9 (16%)	24 (44%)
12-24 months	5 (9%)	10 (18%)
Due to availability and acceptance, I avoid unnecessary colonoscopies. If clinical remission has been achieved, I rely on MRE	38 (69%)	-

Abbreviations: IBD, inflammatory bowel disease; MRE, magnetic resonance enterography.

^a^Results are expressed as numbers and percentages (%).

The survey revealed that 49% of gastroenterologists performed serial mucosal biopsies to assess histological remission in UC patients achieving mucosal healing. This was driven by the perception of potential management implications (33%) or personal interest (16%). However, 25% did not perform serial mucosal biopsies, as they believed it did not impact management. Conversely, 11% reported difficulties in performing mucosal biopsies in patients with mucosal healing due to endoscopists’ reluctance, and 15% due to the fact that pathologists did not use histologic activity indexes.

The survey also addressed transmural healing in patients with ileal CD. Similar to histological remission, 56% of responders carried out serial MRE or ultrasound to monitor transmural healing due to perceived management implications, while 24% did not assess it as they considered that it had minimal management implications. Additionally, 20% were unable to evaluate transmural healing due to a lack of access to MRE or ultrasound.

Finally, 75% of survey participants measured quality of life and other factors such as fatigue, anxiety, sleep quality, or sexuality in daily practice, even though they did not use validated questionnaires. These gastroenterologists aimed to offer adequate support within their means. Conversely, up to 20% of responders believed that many of these aspects were beyond the skills and/or capabilities of gastroenterologists.

### Expert Contributions

Table 6 summarizes barriers and potential solutions for implementing STRIDE-II recommendations according to the experts.

**Table 6.** Barriers to the implementation of STRIDE-II and suggested solutions.

Source	Barrier	Solution
STRIDE-II recommendations	-Lack of robust evidence	- Standardization of definitions and outcomes to facilitate clearer communication between professionals and patients - Research (validation studies, prognosis studies): Promote clinical research focusing on the validation of STRIDE-II targets, particularly in prognostic studies, to provide stronger evidence supporting the recommendations
	-Ambitious or stringent treatment goals (eg, transmural healing)	- Alternative treatment goals: Adopt flexible treatment goals that consider various disease states and patient responses. For example, setting more practical and achievable goals for patients who cannot meet more stringent criteria like transmural healing - Generation of composite scores: Use composite indices that combine several indicators of disease activity to better evaluate patient progress - More flexible cutoffs: Implement less rigid thresholds to account for patient variability in achieving clinical goals, ensuring treatment remains realistic - New drugs and new treatment strategies
	-Scope too general (eg, no subgroups, complex patients)	- Subgroup analysis: Perform detailed subgroup analyses in clinical studies to provide personalized treatment strategies for specific populations (eg, pediatric or elderly patients) - Patient stratification: Stratify patients based on disease severity, comorbidities, and response to previous treatments, allowing for more tailored treatment protocols - Adaptations to specific profiles: Adjust treatment strategies to match individual patient profiles, considering factors like genetics, disease phenotype, and lifestyle
Gastroenterologists	-Lack of knowledge and skills	- Educational activities: Organize continuous medical education (CME) workshops, webinars, and certifications aimed at increasing the awareness and competencies of gastroenterologists regarding STRIDE-II recommendations
	-Lack of time	- Consultation optimization: Streamline patient consultations by incorporating tools like pre-consultation questionnaires or digital health monitoring platforms to ensure efficient data gathering before meetings - Checklists generation: Develop checklists for key clinical steps in implementing STRIDE-II targets, ensuring that important aspects of patient care are not overlooked during time-constrained consultations
Health system	-Poor access (eg, MRE, ultrasound) -Lack of resources	- Improve availability: Advocate for the allocation of more resources and infrastructure that allow for the wider availability of diagnostic tools like MRE and ultrasound in healthcare settings - Local adaptations: Tailor STRIDE-II implementations based on local health system limitations, allowing for the use of available technologies or alternative methods for monitoring disease progress
	-Lack of knowledge and skills in other health professionals (eg, endoscopists)	- Educational activities: Offer specialized training for health professionals such as endoscopists and radiologists, helping them better understand how to apply STRIDE-II targets and accurately interpret diagnostic findings within the framework of IBD care

Abbreviation: MRE, magnetic resonance enterography.

Survey participants rated the importance of STRIDE-II treatment goals as closely linked to the possibility of achieving them in the short- and medium-term. The overall difficulty in accomplishing these goals was high, likely reflecting the chronic, potentially severe, and complex nature of IBD, for which current therapy efficacy may sometimes fall short. However, it could be argued that some of the STRIDE-II treatment goals might be overly stringent and/or lack robust evidence, potentially resulting in suboptimal uptake.^13^

The experts considered that gastroenterologists should ideally aim for disease remission (both clinical and histological). However, recognizing that achieving this last objective may not always be feasible, especially given the limited treatment availability, a pragmatic approach could be adopted in certain scenarios. For those challenging cases, the experts proposed the definition of less stringent goals and/or disease states, similar to those established for other chronic inflammatory conditions such as psoriasis (“almost clear skin,” “disease acceptable state”) and rheumatoid arthritis (“low disease activity,” “good, moderate, or non-responders”).^15^

In the survey, around 25% of respondents indicated that they did not use clinical indices, and 75% did not assess clinical remission through PROs in their daily practice. Several reasons can account for this. Firstly, in CD, clinical symptoms often show a weak correlation with mucosal inflammation.^16^ Besides, functional IBD disorders might be present, impacting the outcomes of subjective variables.^17,18^ Lastly, in busy clinical settings, time constraints may hinder clinical indicator use and/or lead gastroenterologists to prioritize objective measurements over subjective ones.

The experts acknowledged the limitations of clinical indices and PROs, particularly in CD, as well as the time constraints during clinical consultations. However, from the patient’s perspective, clinical symptoms are deemed the most crucial parameters for treatment, and both objective and subjective measures are not mutually exclusive.^19^ Therefore, in daily practice, the experts advocated for the assessment of at least one PRO (preferably a clinical index) in addition to objective variables.

When evaluating FC, most gastroenterologists responded that the first FC assessment occurred around 8-12 weeks after treatment initiation. However, one of the primary survey findings was the response to the question about FC cutoffs. Despite specific cutoff values being stated in the STRIDE-II recommendations for CD and UC, up to 70% of participants chose to monitor changes in FC levels instead of relying on a specific cutoff. This discrepancy could stem from the conflicting evidence associated with FC cutoff values,^20,21^ especially related to their low reliability. FC is considered a non-invasive biomarker contributing to clinical decision-making, as FC reductions have clear prognostic significance.^22,23^ Hence, it should be routinely assessed in daily practice, with relative changes in FC levels also being acknowledged, given the limitations of the proposed cutoffs outlined in the STRIDE-II guidelines.

Three survey questions focused on mucosal healing. Participants indicated that a mucosal evaluation should be conducted in CD patients, regardless of their clinical remission status. This practice may result from the lack of correlation between clinical symptoms and the inflammation degree detected during endoscopy.^24,25^ Interestingly, in cases of clinical remission, MRE was favored over endoscopy. The experts considered that in certain patients with CD limited to small bowel and clinical remission, MRE might be a valid alternative to endoscopy.

Unlike in CD, there is a better correlation between clinical symptoms and endoscopic inflammation in UC.^26^ Adding FC to the clinical evaluation might improve this correlation.^27^ This likely explains why a third of responders did not perform endoscopy in patients showing clinical remission. While we endorse this approach, it is worth noting that endoscopy may also be conducted to rule out functional disorders, address clinical uncertainties, or in patients showing partial responses, among other reasons.

Most respondents concurred that mucosal healing as a long-term treatment goal may not be applicable to all IBD patients, which is in line with findings from the literature.^28^ They also commented that this goal should be carefully tailored, particularly in elderly or fragile patients, in cases of multiple treatment failures, and in last-line treatments, among others. Therefore, individualizing this treatment goal, as currently stated, and adapting it (such as less stringent goals or adjusted timing), or even omitting this treatment goal in specific population subgroups and/or individual patients might be considered. However, as previously noted, mucosal healing should always be considered in every IBD patient.

The survey highlighted various concerns regarding endoscopic scores, including the lack of reliability among some endoscopists and the perceived excessive stringency of the endoscopic cutoff values proposed in the STRIDE-II guidelines for daily practice. Regarding mucosal healing evaluation, we must point out that there is currently no universally accepted definition for mucosal healing in IBD. Additionally, endoscopic response and remission thresholds are inconsistently defined. While the STRIDE-II-proposed endoscopic scores underwent extensive validation, none of these instruments have been fully validated.^29,30^ All of these factors may account for participants’ responses and indicate the need for further research. The experts also emphasized the importance of improving endoscopists’ knowledge and skills regarding IBD and standardizing the endoscopic evaluation and reporting process.^31^ This should include quantifying findings to help therapeutic decision-making.

Regarding histological remission in patients with UC and mucosal healing, approximately half of the participants conducted serial biopsies, with some citing potential management implications as a reason. Although promising data on predicting long-term remission, complications, and cancer have been published,^32^ there is currently no clear evidence supporting its value in therapeutic decision-making. Achieving histological remission in daily practice is highly challenging. However, the experts considered that serial biopsies might be useful for clinical interest and research. On the other hand, histological healing as a therapeutic goal in CD remains challenging. To date, no consensus has been reached to define histological remission and there are no validated indices available.

Most participants performed (or were willing to perform) serial MRE or ultrasound to assess transmural healing in CD patients, primarily due to its management implications. Several studies have shown the association between transmural healing and treatment response as well as long-term outcomes in CD.^33,34^ The advantages of MRE and ultrasound over endoscopy include the ability to perform frequent evaluations and study the entire gastrointestinal tract, including transmural healing, with high patient acceptance. However, achieving transmural healing is challenging with current therapies. Therefore, given the nature of CD, we recommend evaluating transmural healing as a factor to be considered in decision-making, but not as a treatment target. Additionally, further efforts are needed to ensure access to MRE or ultrasound.

Finally, we agree with the majority of survey participants on the importance of prioritizing patients’ quality of life and general well-being.^35,36^

## Discussion

The STRIDE-II recommendations were designed to provide a framework to homogenize the standard for care of IBD patients.^6^

The dissemination of evidence-based recommendations is considered a key step for quality of care improvement. However, simple information dissemination has rarely been effective in changing clinical practices and behavior.^37^ More specifically, in IBD, adherence to and uptake of STRIDE-II recommendations is often suboptimal in real-world settings.^38^

We examined various aspects concerning the implementation of STRIDE-II recommendations, encompassing the advantages and challenges of this approach. We focused on the contextual factors and obstacles to its implementation in real-world clinical practice and put forward potential solutions to address these challenges.

In agreement with other IBD experts,^13^ STRIDE-II recommendations may be challenging to implement to achieve some treatment goals, such as mucosal healing or histological remission, given the limitations of current therapeutic options for IBD patients. These goals may currently be more aspirational than realistic, especially in certain clinical scenarios. However, we maintain that, from a conceptual standpoint, disease remission should always be the main focus of gastroenterologists. Therefore, to overcome this obstacle, we suggest defining and incorporating intermediate (less stringent) treatment goals in challenging scenarios, similar to other diseases such as rheumatoid arthritis (“low disease activity”).^15^

Similarly, some of the proposed measurement tools (including clinical assessments, PROs, biomarkers, imaging, and histological evaluations) for different disease domains (such as activity, disability, and quality of life) may cause reluctance among clinicians, as observed in our study, due to preliminary evidence, lack of validation, or limited applicability or access in daily practice.^16,20,21,24,25,29,30^ For example, although STRIDE-II recommends an FC cutoff of 150 µg/g to identify endoscopic healing, our findings suggest that there is still variability in the implementation of this recommendation, particularly in real-world settings. The reported lack of awareness of clear FC cutoffs by 70% of gastroenterologists may reflect differences in local guidelines, resource availability, or varying levels of familiarity with STRIDE-II among clinicians, especially outside of specialized IBD centers. Further research will be essential to identify standardized, validated, and readily accessible measurement tools. However, in the meantime, it is preferable to utilize these tools for measurement purposes rather than forgoing them altogether.

Another controversial aspect of the STRIDE-II guidelines is IBD simplification or generalization when defining certain treatment goals. Given the vast complexity and heterogeneity of the disease, it is likely that certain patient subtypes, such as elderly patients or those with multiple therapeutic failures, may require a different approach. Consequently, we support the definition of relevant patient profiles and the adaptation of treatment goals and timing accordingly.

We would like to highlight additional obstacles identified in the ANTHEA project, which are often overlooked and involve healthcare professionals and systems. As outlined in our study and observed in other real-world contexts, challenges may arise from the lack of IBD-trained and involved gastroenterologists and endoscopists. Additionally, there may be limited or inadequate access to endoscopy and innovative resources, including advanced imaging techniques and treatments.^38–40^ Other obstacles may also exist, such as financial barriers or time constraints during daily consultations.^38–40^ From an implementation perspective, it is crucial to consider the local context and establish local adaptations and additional implementation strategies to address these issues.

We must acknowledge the limitations of our work. Firstly, we conducted a narrative review rather than a systematic one, which means that there is no guarantee that all relevant articles were identified. However, we utilized the same search techniques employed in systematic reviews, so we can be confident about the adequacy of our review of the evidence. Additionally, the sample size obtained in the survey is relatively small, potentially limiting the result’s generalizability. Nevertheless, we sought to select a diverse sample of gastroenterologists from various regions of Spain, representing different care models and not restricted to academic or IBD centers.

## Conclusions

The STRIDE-II recommendations serve as a valuable framework for guiding the therapeutic management of IBD patients, yet their implementation faces some challenges. Adherence to these guidelines is often suboptimal in real-world settings, requiring a nuanced approach to address barriers such as the challenge of achieving treatment goals and the need for validated measurement tools. Additionally, obstacles related to healthcare professionals and systems, including shortages of trained personnel and limited access to endoscopy and innovative resources, must be considered. Despite limitations in our review methodology and survey sample size, our findings highlight the importance of tailored implementation strategies and ongoing research to enhance the quality of care for IBD patients.

## Data Availability

No new data was created or analyzed.

## References

[1] Alabat S , Sepanlou SG, Ikuta K, et al. The global, regional, and national burden of inflammatory bowel disease in 195 countries and territories, 1990-2017: a systematic analysis for the Global Burden of Disease Study 2017. Lancet Gastroenterol Hepatol. 2020;5(1):17-30. doi:10.1016/s2468-1253(19)30333-4. (In Eng). 31648971 PMC7026709

[2] Bernabeu P , Belén-Galipienso O, van-der Hofstadt C, et al. Psychological burden and quality of life in newly diagnosed inflammatory bowel disease patients. Front Psychol. 2024;15:1334308. doi:10.3389/fpsyg.2024.1334308. (In eng). 38348263 PMC10859525

[3] Saleh A , Abraham BP. Utility of intestinal ultrasound in clinical decision-making for inflammatory bowel disease. Crohns Colitis 360. 2023;5(3):otad027. doi:10.1093/crocol/otad027. (In eng) 37292105 PMC10246583

[4] Ashraf H , Bodapati A, Hanif A, et al. Safety and efficacy of biologic therapies (ustekinumab and vedolizumab) in the treatment of Inflammatory Bowel Disease (IBD): a systematic review. Cureus. 2023;15(11):e48338. doi:10.7759/cureus.48338. (In eng) 38060699 PMC10698389

[5] Solitano V , Vuyyuru SK, MacDonald JK, et al. Efficacy and safety of advanced oral small molecules for inflammatory bowel disease: systematic review and meta-analysis. J Crohns Colitis. 2023;17(11):1800-1816. doi:10.1093/ecco-jcc/jjad100. (In eng) 37317532

[6] Turner D , Ricciuto A, Lewis A, et al.; International Organization for the Study of IBD. STRIDE-II: an update on the Selecting Therapeutic Targets in Inflammatory Bowel Disease (STRIDE) Initiative of the International Organization for the Study of IBD (IOIBD): determining therapeutic goals for treat-to-target strategies in IBD. Gastroenterology. 2021;160(5):1570-1583. doi:10.1053/j.gastro.2020.12.031. (In eng) 33359090

[7] Geyl S , Guillo L, Laurent V, D’Amico F, Danese S, Peyrin-Biroulet L. Transmural healing as a therapeutic goal in Crohn’s disease: a systematic review. Lancet Gastroenterol Hepatol. 2021;6(8):659-667. doi:10.1016/S2468-1253(21)00096-0. (In eng) 34090579

[8] Shah SC , Colombel JF, Sands BE, Narula N. Mucosal healing is associated with improved long-term outcomes of patients with ulcerative colitis: a systematic review and meta-analysis. Clin Gastroenterol Hepatol. 2016;14(9):1245-1255.e8. doi:10.1016/j.cgh.2016.01.015. (In eng) 26829025

[9] Alayo QA , Fenster M, Altayar O, et al. Systematic review with meta-analysis: safety and effectiveness of combining biologics and small molecules in inflammatory bowel disease. Crohns Colitis 360. 2022;4(1):otac002. doi:10.1093/crocol/otac002. (In eng) 35310082 PMC8924906

[10] Colombel JF , D’Haens G, Lee WJ, Petersson J, Panaccione R. Outcomes and strategies to support a treat-to-target approach in inflammatory bowel disease: a systematic review. J Crohns Colitis. 2020;14(2):254-266. doi:10.1093/ecco-jcc/jjz131. (In eng) 31403666 PMC7008150

[11] Peyrin-Biroulet L , Sandborn W, Sands BE, et al. Selecting Therapeutic Targets in Inflammatory Bowel Disease (STRIDE): determining therapeutic goals for treat-to-target. Am J Gastroenterol. 2015;110(9):1324-1338. doi:10.1038/ajg.2015.233. (In eng) 26303131

[12] Herrlinger KR , Stange EF. To STRIDE or not to STRIDE: a critique of “treat to target” in Crohn’s disease. Expert Rev Gastroenterol Hepatol. 2023;17(12):1205-1219. doi:10.1080/17474124.2023.2296564. (In eng) 38131269

[13] Dignass A , Rath S, Kleindienst T, Stallmach A. Review article: translating STRIDE-II into clinical reality—opportunities and challenges. Aliment Pharmacol Ther. 2023;58(5):492-502. doi:10.1111/apt.17622. (In eng) 37382397

[14] CEBM., Medicine. CfEB. CEBM Levels of Evidence 2011. University of Oxford; 2011. http://www.cebm.net/index.aspx?o=1025

[15] Smolen JS , Landewé RBM, Bergstra SA, et al. EULAR recommendations for the management of rheumatoid arthritis with synthetic and biological disease-modifying antirheumatic drugs: 2022 update. Ann Rheum Dis. 2023;82(1):3-18. doi:10.1136/ard-2022-223356. (In eng) 36357155

[16] Laterza L , Piscaglia AC, Minordi LM, et al. Multiparametric evaluation predicts different mid-term outcomes in Crohn’s disease. Dig Dis. 2018;36(3):184-193. doi:10.1159/000487589. (In eng) 29514146

[17] Farrokhyar F , Marshall JK, Easterbrook B, Irvine EJ. Functional gastrointestinal disorders and mood disorders in patients with inactive inflammatory bowel disease: prevalence and impact on health. Inflamm Bowel Dis. 2006;12(1):38-46. doi:10.1097/01.mib.0000195391.49762.89. (In eng) 16374257

[18] Bryant RV , van Langenberg DR, Holtmann GJ, Andrews JM. Functional gastrointestinal disorders in inflammatory bowel disease: impact on quality of life and psychological status. J Gastroenterol Hepatol. 2011;26(5):916-923. doi:10.1111/j.1440-1746.2011.06624.x. (In eng) 21214889

[19] Peek-Kuijt NMS , Aantjes MJ, Verwey M, Van Bodegom-Vos L, van der Meulen-de Jong AE, Maljaars JPW. Treatment goals in IBD: a perspective from patients and their partners. PEC Innov. 2022;1:100034. doi:10.1016/j.pecinn.2022.100034. (In eng) 37213759 PMC10194327

[20] Rokkas T , Portincasa P, Koutroubakis IE. Fecal calprotectin in assessing inflammatory bowel disease endoscopic activity: a diagnostic accuracy meta-analysis. J Gastrointestin Liver Dis. 2018;27(3):299-306. doi:10.15403/jgld.2014.1121.273.pti. (In eng) 30240474

[21] Guidi L , Marzo M, Andrisani G, et al. Faecal calprotectin assay after induction with anti-Tumour Necrosis Factor α agents in inflammatory bowel disease: prediction of clinical response and mucosal healing at one year. Dig Liver Dis. 2014;46(11):974-979. doi:10.1016/j.dld.2014.07.013. (In eng) 25096964

[22] Schoepfer AM , Beglinger C, Straumann A, Trummler M, Renzulli P, Seibold F. Ulcerative colitis: correlation of the Rachmilewitz endoscopic activity index with fecal calprotectin, clinical activity, C-reactive protein, and blood leukocytes. Inflamm Bowel Dis. 2009;15(12):1851-1858. doi:10.1002/ibd.20986. (In eng) 19462421

[23] Sollelis E , Quinard RM, Bouguen G, et al. Combined evaluation of biomarkers as predictor of maintained remission in Crohn’s disease. World J Gastroenterol. 2019;25(19):2354-2364. doi:10.3748/wjg.v25.i19.2354. (In eng) 31148906 PMC6529885

[24] Tse CS , Singh S, Valasek MA, et al. Prevalence and correlations of gastrointestinal symptoms with endoscopic and histologic mucosal healing in Crohn’s disease. Am J Gastroenterol. 2023;118(4):748-751. doi:10.14309/ajg.0000000000002122. (In eng) 36623171

[25] Lewis JD , Rutgeerts P, Feagan BG, et al. Correlation of stool frequency and abdominal pain measures with simple endoscopic score for Crohn’s disease. Inflamm Bowel Dis. 2020;26(2):304-313. doi:10.1093/ibd/izz241. (In eng) 31644790

[26] Arias MT , Vande Casteele N, Vermeire S, et al. A panel to predict long-term outcome of infliximab therapy for patients with ulcerative colitis. Clin Gastroenterol Hepatol. 2015;13(3):531-538. doi:10.1016/j.cgh.2014.07.055. (In eng) 25117777

[27] Zittan E , Kelly OB, Kirsch R, et al. Low fecal calprotectin correlates with histological remission and mucosal healing in ulcerative colitis and colonic Crohn’s disease. Inflamm Bowel Dis. 2016;22(3):623-630. doi:10.1097/mib.0000000000000652. (In eng) 26829408

[28] Cucchiara S , D’Arcangelo G, Isoldi S, Aloi M, Stronati L. Mucosal healing in Crohn’s disease: new insights. Expert Rev Gastroenterol Hepatol. 2020;14(5):335-345. doi:10.1080/17474124.2020.1759416. (In eng) 32315209

[29] Mohammed Vashist N , Samaan M, Mosli MH, et al. Endoscopic scoring indices for evaluation of disease activity in ulcerative colitis. Cochrane Database Syst Rev. 2018;1(1):Cd011450. doi:10.1002/14651858.CD011450.pub2. (In eng) 29338066 PMC6491285

[30] Khanna R , Nelson SA, Feagan BG, et al. Endoscopic scoring indices for evaluation of disease activity in Crohn’s disease. Cochrane Database Syst Rev. 2016;2016(8):Cd010642. doi:10.1002/14651858.CD010642.pub2. (In eng) 27501379 PMC7079710

[31] Maaser C , Sturm A, Vavricka SR, et al.; European Crohn’s and Colitis Organisation [ECCO] and the European Society of Gastrointestinal and Abdominal Radiology [ESGAR]. ECCO-ESGAR Guideline for Diagnostic Assessment in IBD Part 1: initial diagnosis, monitoring of known IBD, detection of complications. J Crohns Colitis. 2019;13(2):144-164K. doi:10.1093/ecco-jcc/jjy113. (In eng) 30137275

[32] Mojtahed A , Khanna R, Sandborn WJ, et al. Assessment of histologic disease activity in Crohn’s disease: a systematic review. Inflamm Bowel Dis. 2014;20(11):2092-2103. doi:10.1097/mib.0000000000000155. (In eng) 25137418

[33] Fernandes SR , Rodrigues RV, Bernardo S, et al. Transmural healing is associated with improved long-term outcomes of patients with Crohn’s disease. Inflamm Bowel Dis. 2017;23(8):1403-1409. doi:10.1097/mib.0000000000001143. (In eng) 28498158

[34] Deepak P , Fletcher JG, Fidler JL, et al. Predictors of durability of radiological response in patients with small bowel Crohn’s disease. Inflamm Bowel Dis. 2018;24(8):1815-1825. doi:10.1093/ibd/izy074. (In eng) 29668921 PMC6391864

[35] Barreiro-de Acosta M , Marín-Jiménez I, Panadero A, et al. Recommendations of the Spanish Working Group on Crohn’s Disease and Ulcerative Colitis (GETECCU) and the Association of Crohn’s Disease and Ulcerative Colitis Patients (ACCU) in the management of psychological problems in inflammatory bowel disease patients. Gastroenterol Hepatol. 2018;41(2):118-127. doi:10.1016/j.gastrohep.2017.10.003. (In eng spa) 29275001

[36] Casellas F , Guinard Vicens D, García-López S, et al. Consensus document on the management preferences of patients with ulcerative colitis: points to consider and recommendations. Eur J Gastroenterol Hepatol. 2020;32(12):1514-1522. doi:10.1097/MEG.0000000000001885. (In eng) 32804838

[37] Schectman JM , Schroth WS, Verme D, Voss JD. Randomized controlled trial of education and feedback for implementation of guidelines for acute low back pain. J Gen Intern Med. 2003;18(10):773-780. doi:10.1046/j.1525-1497.2003.10205.x. (In eng) 14521638 PMC1494929

[38] Vega P , Huguet JM, Gómez E, et al. IBD-PODCAST Spain: a close look at current daily clinical practice in IBD management. Dig Dis Sci. 2024;69(3):749-765. doi:10.1007/s10620-023-08220-9. (In eng) 38217680 PMC10960747

[39] Watermeyer G , Katsidzira L, Nsokolo B, et al. Challenges in the diagnosis and management of IBD: a sub-Saharan African perspective. Therap Adv Gastroenterol. 2023;16:17562848231184986. doi:10.1177/17562848231184986. (In eng) PMC10345935 37457138

[40] Devi J , Deepak P, Butt AS. Strategies for enhancing inflammatory bowel disease care in Pakistan: bridging gaps and building capacities. Crohns Colitis 360. 2024;6(2):otae027. doi:10.1093/crocol/otae027. (In eng) 38720936 PMC11078041

